# Evolutionary inferences from the analysis of mutation dynamics in the SARS-CoV-2 replication-transcription complex

**DOI:** 10.18699/vjgb-26-54

**Published:** 2026-05

**Authors:** A.Yu. Palyanov, A.P. Devyaterikov, N.V. Palyanova, A.M. Shestopalov

**Affiliations:** A.P. Ershov Institute of Informatics Systems of the Siberian Branch of the Russian Academy of Sciences, Novosibirsk, Russia Research Institute of Virology, Federal Research Center of Fundamental and Translational Medicine, Novosibirsk, Russia; A.P. Ershov Institute of Informatics Systems of the Siberian Branch of the Russian Academy of Sciences, Novosibirsk, Russia; Research Institute of Virology, Federal Research Center of Fundamental and Translational Medicine, Novosibirsk, Russia; Research Institute of Virology, Federal Research Center of Fundamental and Translational Medicine, Novosibirsk, Russia

**Keywords:** SARS-CoV-2, RdRp, RTC, evolution, substitutions, mutations, dynamics, analysis, nsp12:G671S, Delta, SARS-CoV-2, RdRp, RTC, эволюция, замены, мутации, динамика, анализ, nsp12:G671S, Дельта

## Abstract

The SARS-CoV-2 virus continues to evolve and remains a significant public health threat, while the worldwide monitoring and sequencing of its genomic variants provide a unique opportunity to study its evolution and better understand its molecular mechanisms. In our work, we analyze its replication-transcription complex (RTC) over a 5.5- year period (December 2019–July 2025). This complex is significantly more conserved (as any alteration impairing its function prevents viral replication) than the S-protein (directly impacting infectivity and immune evasion) but still dynamically evolving part of the genome. The study focuses on high-frequency substitutions, their temporal behavior, co-occurrence, and structural context. Using genomes from GISAID, we identified 22 amino acid point mutations present in at least 1 % of currently available sequences, analyzed their weekly dynamics, revealed three distinct temporal patterns, and enumerated frequent co-occurring groups (pairs, triplets, and larger sets) within the same genomes. We mapped the affected residues onto an RTC 3D structure and reviewed the literature to examine the reported functional consequences. Notably, all these substitutions were single-nucleotide. One of the mutations, nsp12:G671S, showed a unique dynamic feature: it emerged, dominated globally for months, disappeared twice, and in 2025 reappeared for the 3rd time, always accompanied with other mutations in the RTC. Thus, it was interesting to trace its dynamics as an indicator of probable changes. In addition, our analysis of mutation and variant timelines suggests that the Delta variant may have emerged 7–8 months earlier than commonly reported. Taken together, these results provide a consolidated view of recurrent RTC variation, its temporal classes, co-occurrence, and structural context, underscoring the value of systematic surveillance of nsp7–nsp14 alongside analyses focused on structural proteins.

## Abstract (ru)

Вирус SARS-CoV-2 остается заметной угрозой для человечества, поскольку продолжает циркулировать и эволюционировать. При этом продолжающийся глобальный мониторинг и накопление геномных данных позволяют детально изучать эволюционные механизмы. В нашей работе мы анализируем репликационно-транскрипционный комплекс (RTC, nsp7–nsp14) – более консервативную, чем S-белок, напрямую связанный с инфекционностью и избеганием иммунитета, но все-таки динамично эволюционирующую часть генома – в течение 5.5-летнего периода (декабрь 2019 г. – июль 2025 г.). Исследование сосредоточено на аминокислотных заменах, присутствующих по крайней мере в 1 % доступных в базе GISAID геномов, их временнóй динамике, совместной встречаемости и структурном контексте. Мы идентифицировали 22 таких точечных аминокислотных замены, проанализировали их недельную динамику, выявили три различных временн’ых паттерна и рассчитали частоты для групп мутаций (пары, тройки и т. д.), одновременно присутствующих в геномах. Примечательно, что все изученные замены были однонуклеотидными. Мы визуализировали аминокислотные остатки, соответствующие рассмотренным мутациям, на трехмерной структуре RTC, описали их особенности и обобщили данные литературы для изучения известных функциональных последствий. Одна из мутаций, nsp12:G671S, продемонстрировала уникальную динамику: она появлялась, доминировала в глобальном масштабе в течение нескольких месяцев, затем исчезала дважды, а в 2025 г. появилась в третий раз. При этом она всегда сопровождалась набором сопутствующих мутаций в комплексе RTC, что делает ее потенциальным индикатором изменений в геноме, т. е. необходимо продолжить отслеживание ее динамики. Кроме того, наш анализ временны’ х линий мутаций и вариантов позволяет предположить, что вариант Дельта мог появиться на 7–8 месяцев раньше, чем принято считать. В совокупности эти результаты дают целостное представление о повторяющихся вариациях в RTC, их временны’ х классах и совместной встречаемости, а также о структурном контексте, подчеркивая ценность систематического мониторинга nsp7–nsp14 наряду с анализом, сфокусированным на структурных белках.

## Introduction

The COVID-19 pandemic catalyzed a sustained global effort in next-generation sequencing, virology, and bioinformatics, yielding an unprecedented corpus of SARS-CoV-2 genomes over the past five years. As the virus continues to evolve and remains a major public health concern, continuous genomic surveillance and further investigation of its molecular mechanisms are of considerable importance. The 29.9-kb positive-sense RNA genome encodes structural (S, E, M, N), nonstructural (nsp1–nsp16), and nine small accessory proteins (Bai et al., 2022; Yan W. et al., 2022), with approximately two-thirds of the coding capacity assigned to the nonstructural machinery on the 5′ end (Eriani, Martin, 2022). A similar genomic structure, in terms of both gene composition and gene order, is observed across multiple related coronaviruses, including those hosted by humans, bats, and pangolins (Brant et al., 2021, Fig. 1; Temmam et al., 2022, Fig. 1). Approximately two-thirds of the genome on the 5′ part codes for non-structural proteins and one-third of the genome on the 3′ part codes for structural and accessory proteins (Eriani, Martin, 2022).

**Fig. 1.**
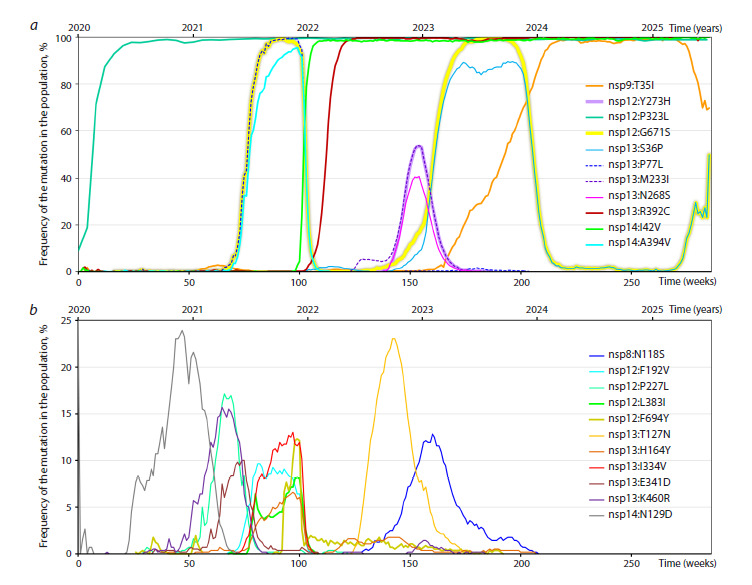
Global frequency dynamics of mutations within RTC. Weekly frequencies of 22 substitutions in the RTC proteins (listed in Table 1) from January 2020 to July 2025. a, Trajectories of mutations that reached a peak frequency between 25 and 100 %. b, Trajectories of mutations with a peak frequency below 25 %. Frequencies were calculated as the proportion of all GISAID genomes per week containing a specific mutation.

Mutations in the S-protein gene (~13 % of the 29.9 kb genome) attract the greatest attention due to their direct impact on infectivity and immune evasion. However, mutations in other structural and nonstructural proteins, as well as recombination events and other genomic alterations, can also lead to diverse consequences that affect virus adaptability. SARS-CoV-2 exhibits a complex interplay between antigenicity, transmissibility, and virulence, which usually has unpredictable consequences for the future trajectory of evolution of the virus (Carabelli et al., 2023). In the present study, we continue our investigation of SARS-CoV-2 evolution, using genomic sequences that have been collected and deposited in public databases since the virus first emerged in late 2019. In our previous works, we analyzed SARS-CoV-2 evolution at several scales: globally (Palyanov, Palyanova, 2024), regionally across Siberia (Palyanova et al., 2023), and within the genomic variant landscape of the virus (Palyanov, Palyanova, 2023). Here, we focus on a mutational analysis of the replication-transcription complex, which carries out several essential viral functions, including genome replication and proofreading, and constitutes a substantial portion of the viral genome (Romano et al., 2020).

Among nonstructural proteins, the replication-transcription complex (RTC) is central to RNA synthesis and error control. The nsp12 RNA-dependent RNA polymerase (RdRp) operates with cofactors nsp7 and nsp8. Together they form a subcomplex (holoenzyme), which is the minimal core component for mediating coronavirus RNA synthesis (Peng et al., 2020; Singh et al., 2025). In this work we analyze an extended, structurally characterized RTC assembly spanning nsp7–nsp14 (PDB: 7EIZ) (Yan L. et al., 2021), which enables assessment of substitutions not only within RdRp but also across its binding interfaces and the exonuclease proofreading module. The analysis of this extended configuration may provide additional insights into the structural and functional consequences of mutations, particularly in cases where multiple substitutions occur in close spatial proximity. For example, point mutations at specific sites of the nsp12–nsp8 interface dramatically affect the RNA polymerization activity of SARS-CoV-2 (Ferrer-Orta et al., 2024). In the same year 2024, it was discovered that the combination of nsp8:A21V and nsp12:P323L mutations resulted in an approximately 50 % increase in polymerase activity (Danda et al., 2024). The authors described this as the first biochemical study demonstrating the functional impact of amino acid substitutions involving all components of the RdRp complex in emerging SARS-CoV-2 subvariants.

The evolution of SARS-CoV-2 proceeds through a mixture of point mutations, insertions, deletions, and recombinations, and its consequences are highly diverse and difficult to predict. For instance, mutations in nonstructural proteins such as nsp6:ΔSGF(3675–3677) (Feng et al., 2023) and nsp12:P323L/ G671S (Kim et al., 2023) have been shown to increase viral replication efficiency. Next, nsp1:(Y154A/F157A) and nsp1:(R171E/R175E) mutations have been found to abolish protein translation inhibition in a cell free system (Schubert et al., 2020). The single mutation nsp14:F60S in the exoribonuclease (ExoN), responsible for replication proofreading, accelerates viral evolution by increasing the mutation rate (Mack et al., 2023). Moreover, the evolutionary rate of SARSCoV- 2 varies considerably among variants, and the emergence of new lineages often coincides with episodic accelerations of this rate. In certain periods, the evolutionary rate increased up to fourfold relative to the background phylogenetic rate, leading to the emergence of new variants within weeks rather than months, as would be expected from the baseline tempo of viral evolution (Tay et al., 2022).

While early global surveys mapped common substitutions across the genome (Abbasian et al., 2023), a focused and timeresolved analysis of RTC variation at population scale is still warranted given its functional importance. Genes encoding RTC are among the most conserved in viral genomes, as malfunction of the replication machinery prevents the production of viable virions. Nevertheless, SARS-CoV-2 (2019) exhibits four to eight point amino acid substitutions compared to its closest relatives (e. g., BANAL-20-52 (Temmam et al., 2022; Ou et al., 2023) and RaTG13 (Rahalkar, Bahulikar, 2020; Zhou et al., 2020)), a range similar to the divergence observed between the RTC genes of the ancestral SARS-CoV-2 and of a typical 2023 Omicron descendant (Table S1)1. The RNAdependent RNA polymerases (RdRps) of viruses and cellular organisms share a conserved structural core, most notably the canonical palm domain that forms the catalytic center of the polymerase, underscoring the universality of the RNA synthesis mechanism. However, they diverge significantly in their structural details and a variety of accessory proteins – so, there is scope for variation, and the virus exploits it during evolution. Therefore, it is crucial to monitor the rate and consequences of these changes to identify emerging, functionally significant RTC substitutions that rise above the background noise in global surveillance data.

Supplementary Materials are available in the online version of the paper: https://vavilovj-icg.ru/download/pict-2026-30/appx28.pdf


In this study, we aimed to identify RTC substitutions that eventually attain population-level peaks and to characterize their post-peak behaviors, including distinct cycles of reemergence and decline or extinction, while also assessing co-occurrence within the extended complex. Using all publicly available genomes from late 2019 to July 2025, we quantified amino acid substitutions in nsp7–nsp14 occurring in ≥1 % of sequences; profiled their weekly and monthly dynamics to classify persistent, transient, and recurrent patterns; and analyzed co-occurrence. We then interpreted these populationscale signals in structural and functional terms by centering the analysis on the structurally resolved RTC (nsp7–nsp14; PDB 7EIZ), with the broader genome-wide context provided by prior surveys (Abbasian et al., 2023).

## Materials and methods

We performed a secondary analysis of SARS-CoV-2 genomic surveillance data in the public domain to quantify the amino acid (AA) substitution dynamics in the proteins of the replication- transcription complex (RTC: nsp7/8/9/10/12/13/14) from the start of the pandemic through July 15, 2025. The primary outcome was the weekly global fraction of genomes carrying a given AA substitution; secondary outcomes included (i) geographic stratification by continent, (ii) co-occurrence patterns among substitutions within RTC genes, and (iii) lineage/clade context for substitutions with notable temporal behavior. The pre-specified gene set and date horizon are described in the main text and the Results sections

**Data sources**

1. GISAID (https://gisaid.org) (Khare et al., 2021): the source of genome sequences and metadata used for lineage context and targeted queries by mutation, date, and location. All GISAID queries used the web interface (EpiCov→Search) with the filters complete + low coverage excluded + collection date complete for reliability, as detailed in the Results and Materials sections of the manuscript. It retains 15.5 of 17.5 million of genome samples, whereas the stronger variant, high coverage instead of low coverage excluded (entries with <1 % of Ns vs entries with <5 % of Ns), keeps only 5.7 million genomes. Text input fields for collection ( from) and collection (to) dates were used to specify the necessary time interval, AA Substitutions, to input single or multiple mutations in proteins (for example, nsp9_T35I, nsp12_G671S, nsp13_S36P), and Nucl. Mutations, to input single or multiple mutations in genome nucleotides.

2. CovidCG (https://covidcg.org) (Elbe, Buckland-Merrett, 2017): weekly counts of genomes carrying specified AA substitutions, and tools for comparing/combining substitutions within a gene (the Compare AA mutations module), used to obtain mutation frequency time-series and co-occurrence tallies. As of July 15, 2025, CovidCG reported 21,082,039 analyzable genomes (vs. 17,413,645 in the GISAID’s own counter).

3. Nextclade (https://clades.nextstrain.org) (Aksamentov et al., 2021): used for clade assignment and recombinant flags on downloaded FASTA selections.

4. PDB structure 7EIZ from the Protein Data Bank, PDB (https://www.rcsb.org) and PyMOL v3.1 (https://www. pymol.org) were used solely for structure-mapping of residues corresponding to substitutions.

5. SARS-CoV-2 (COVID-19) stores the genome map of the Wuhan-Hu-1 isolate, with complete nucleotide and amino acid sequences (https://www.snapgene.com/plasmids/ coronavirus_resources/SARS-CoV-2_(COVID-19)_ Genome); together with GISAID’s genome sequences lists of nucleotide and corresponding amino acid mutations available for all genome samples. We used it to create the table, showing which specific codon triplets in nucleotide sequences represent protein sequences in the reference genome

**Inclusion criteria and quality control**

Sequences: human SARS-CoV-2 genomes flagged as complete, with low-coverage entries excluded, and complete collection dates (YYYY-MM-DD) required. These exact GISAID web interface options are enumerated in the Results/Methods narrative and were applied to every targeted query (e. g., first occurrence tables).

Time frame: December 2019 – July 15, 2025, matching the scope stated in the paper. Access dates for all web tools were within July 1–15, 2025 (final extraction and checks on July 15, 2025). All queries (filters, date ranges, and mutation lists) are specified in the main text (Tables/Figures).

Gene set: RTC proteins nsp7/8/9/10/12/13/14.

**Data acquisition for single-point mutation frequencies**

Data on amino acid substitution frequencies in SARS-CoV-2 RTC proteins (nsp7–nsp14) were obtained from the Compare AA Mutations section of the CovidCG platform (https:// covidcg.org). This tool provides aggregated daily/weekly/ monthly/annual counts of genomes carrying specific amino acid substitutions based on submissions to the GISAID database.

For each RTC protein (nsp7 through nsp14), amino acid– level data were selected. The grouping parameter was set to mutation, and the genomic coordinate system defined by protein. The analysis was performed using the full residuerange for each protein and included all geographic regions. The time range was set to cover the entire pandemic period (from December 2019 to July 2025) according to the Since pandemic start preset.

We extracted the information on all single amino acid substitutions with a total global frequency exceeding 1 % from the resulting datasets. This threshold is not a biological or statistical boundary. It was chosen as a pragmatic, operational cutoff to suppress locality-driven and unstable low-frequency noise and to keep the population-scale analysis tractable. Functionally important sub-1 % changes can exist, but these fall outside the scope of our population-scale focus. For each mutation, the CovidCG interface provides the number and proportion of genomes carrying the substitution relative to the total number of genomes available for the corresponding week, and the figures were used in further analysis of temporal dynamics.

**Construction of weekly frequency-time series**

Building on the dataset described above, we investigated the weekly dynamics of each identified mutation using the AA Mutation Co-occurrence module of the mentioned CovidCG platform. For each selected amino acid substitution within RTC proteins (nsp7–nsp14), we examined the New AA Mutation Percentages by Week chart, which displays the proportion of SARS-CoV-2 genomes carrying a given substitution over time. The data are shown as percentages and grouped by week to visualize the mutation prevalence dynamics throughout the entire observation period. The resulting time series for each substitution were exported via the CovidCG Download function and used to assess temporal trends in mutation frequencies.

**Classification of dynamic types**

Each mutation identified within the RTC (nsp7–nsp14) was represented as a weekly series reflecting the proportion of genomes carrying that substitution relative to the total number of genomes reported globally for the same week.

The observed temporal trajectories were categorized into three distinct dynamic types based on their characteristic shapes and relative frequency changes over time.

Persistent or fixation-like pattern. The frequency of the mutation exhibited a rapid increase from near 0 % to ≥90–100 %, followed by a consistently high level for the remainder of the observation period. These trajectories correspond to mutations that became fixed or near-fixed in the global SARS-CoV-2 population.

Transient pattern. The mutation frequency increased noticeably (typically reaching 10–60 %) but subsequently declined to near 0 %, indicating the rise and disappearance of a temporary lineage or variant in which the substitution was predominant.

Recurrent pattern. The mutation frequency showed multiple distinct peaks separated by intervals of decline, i. e., a pattern of emergence, near-complete disappearance, and subsequent re-establishment of high frequency (≥80–100 %). In our dataset, this behavior was observed only for the nsp12:G671S substitution. This mutation was accompanied by different sets of co-occurring substitutions during individual peaks, each exhibiting highly correlated frequency profiles throughout their respective intervals.

**Lineage and clade context**

Nextclade was run on representative FASTA samples exported from the GISAID for periods of rising and falling phases to obtain Nextstrain clades and recombinant flags. The Pango lineage labels shown in tables/figures were taken from the GISAID metadata of the same records (e. g., B.1.617.2/Delta, XBB.*, JN.1, etc.).

**Structural mapping**

Substitutions with a weekly peak frequency of over 25 % were mapped onto 7EIZ (extended RTC) with residue atoms rendered as spheres in PyMOL v3.1 (The PyMOL Molecular Graphics System, Version 3.1 Schrödinger, LLC, https:// www.pymol.org). Figures were rendered directly from these models and used solely to visualize the spatial dispersion and proximity to RNA. No structural inference beyond visualization was performed.

## Results and discussion

**Mutations in SARS-CoV-2 RTC (nsp7–nsp14) proteins with overall average frequencies >1 % in the population**

The list of mutations in nsp7–nsp14 proteins with the total average frequency exceeding 1 % in the global dataset during the period from January 1, 2020 to July 15, 2025 was obtained from CovidCG.org (Elbe, Buckland-Merrett, 2017), as described in “Data acquisition for single-point mutation frequencies” (section Materials and Methods). The results are presented in Table 1. The rightmost column contains values of peak weekly frequency of the same mutations, obtained as described in “Construction of weekly frequency-time series” (section Materials and Methods). In total, 22 mutations were identified.

**Table 1.**
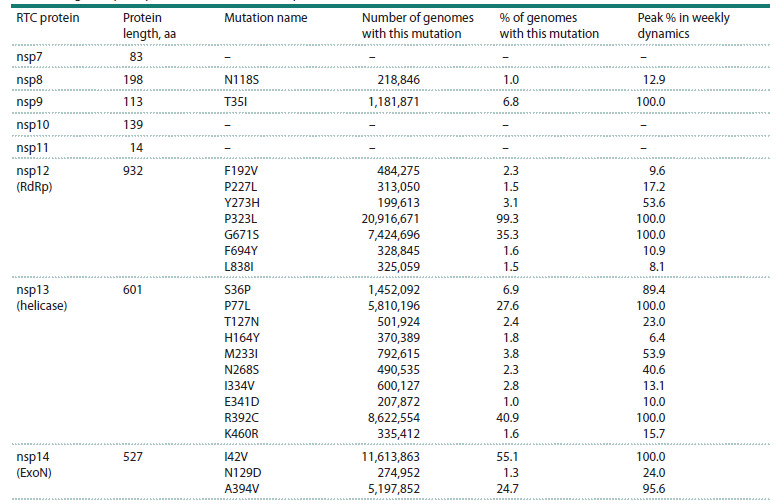
High-frequency substitutions in the RTC proteins Note. Substitutions present in at least 1 % of genomes were collected globally for the period from December, 2019 to July 15, 2025. For each mutation, the number of genomes with this mutation, the percentage of genomes with this mutation in the global population, and the weekly peak percentage are presented.

**Dynamics of SARS-CoV-2 mutation frequencies within RTC**

The data on the weekly dynamics of mutation frequencies (from CovidCG.org) presented in Table 1 were obtained as described in “Construction of weekly frequency-time series” (section Materials and Methods) . The analyzed time interval is from January 1, 2020 to July 15, 2025.

We classified the curves presented in Figure 1 into three dynamic patterns: (1) persistent or fixation-like, (2) transient and (3) recurrent, as described in “Classification of dynamic types”. We noticed only one mutation out of 22, nsp12:G671S, with two specific properties that distinguished it from all the others

First, upon its emergence, it reached a 100 % frequency, remained dominant for months and then disappeared; however, the cycle repeated, and the mutation appears to be undergoing this process for the third time at present. Second, during each round of emergence, it was accompanied by other RTC mutations during either a single tide (nsp13:S36P, nsp14:A394V) or two subsequent tides (nsp13:S36P). Their mutation frequency curves during this period also reach high values (up to 90–100 %) and closely resemble that of nsp12:G671S.

**The dynamics of nsp12:G671S frequency in the population – two and a half tides**

*The first tide of nsp12:G671S*

The atypical dynamics of nsp12:G671S frequency in the population showed two complete “tides” (by analogy with the SARS-CoV-2 pandemic waves), each consisting of an emergence phase, growth, a plateau, and a decline to near-zero level. Early in 2025, a third tide appeared to have begun, and it is still ongoing. As we needed to better understand the process dynamics, we conducted an investigation to determine the connection between these events and the dominance periods of the Delta, Omicron, and other variants.

**Fig. 2.**
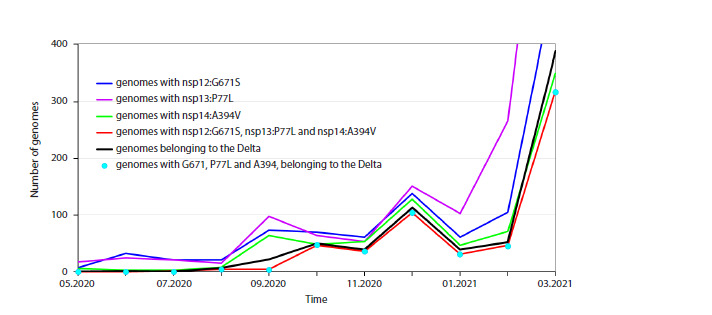
Dynamics of the number of genomes with three key mutations in RTC genes related to the Delta variant. Shortly after their simultaneous appearance in the Delta genomes, their number started to increase rapidly. Monthly numbers of genomes (GISAID) carrying only one of the mutations (nsp12:G671S, nsp13:P77L or nsp14:A394V), genomes with all three mutations and genomes belonging to Delta variant during the latent period (less than 10 cases per month) before the apparent rise of Delta in May 2021 are shown

Since the frequency curves of genomes with nsp12:G671S, nsp13:P77L and nsp14:A394V almost completely overlapped during the first tide (May 2021 – May 2022), rising from near 0 % to nearly 100 % level and back, it is highly probable that these three mutations tended to occur together. Indeed, among 4.12 million genomes with nsp12:G671S, 4.19 million with nsp13:P77L and 3.81 million with nsp14:A394V (collection dates between May 1, 2021 and May 1, 2022 in GISAID), 3.75 million contained all the three amino acid substitutions simultaneously. Moreover, all samples collected in mid-May 2021 (during the rise of the tide) belonged to the Pango lineage B.1.617.2+AY.*, also known as the Delta variant. Our analysis aimed to determine whether the genomes carrying the nsp12:G671S + nsp13:P77L + nsp14:A394V combination or the Delta variant appeared first, or whether they appeared simultaneously. The results indicate that the latter is likely. Supporting details and evidence for this conclusion are shown in Figure 2

By March 2020, the SARS-CoV-2 genome variants carrying nsp12:G671S, nsp13:P77L, and nsp14:A394V already existed. However, according to GISAID data, none of the samples contained them all simultaneously. When this combination first appeared in August 2020, the earliest genome identified as belonging to the Delta variant was detected, and it carried precisely these three mutations. Nearly all Delta genomes collected and sequenced thereafter also contained this mutation trio, suggesting that these mutations may have been required for the emergence and/or fitness advantage of Delta. Literature analysis revealed that this mutation trio had been mentioned in two articles describing variants detected in Iran (Ahmadi et al., 2023) and India (Bokolia, Gadepalli, 2022), but apparently received little attention at the time.

*Timing the emergence of the Delta variant*

The analysis of samples corresponding to the Delta variant (including early events classified as B.1.617.2 / Delta based on GISAID Pango metadata) and containing the trio of mutations in question led us to an accidental finding: according to these data the Delta variant may have emerged 7–8 months earlier than commonly reported in the literature, and in a different geographical location. This observation is based on the GISAID data, which includes hundreds of genome samples (filtered by complete + low coverage excluded + collection date complete for reliability). Detailed data providing the evidence and explaining this conclusion are presented in the Supplementary Materials, Table S2 and Figure S1. In brief, the earliest B.1.617.2 (Delta) genome was collected on April 26, 2020 in “Europe/Germany/Freiburg” (GISAID EPI_ISL=852796). This contradicts the statement that “The Delta (B.1.617.2) variant was first identified in Maharashtra, India, in late 2020” (Cherian et al., 2021), which is frequently cited in earlier (Mlcochova et al., 2021), later (Rahman et al., 2023), and very recent (Kandhasamy et al., 2025) publications.

According to GISAID data, during the period from April 26, 2020 to December 9, 2020 – that is, before the first Delta case reported in India (Maharashtra) in “late 2020” (December 10, 2020, GISAID EPI_ISL_2131509) – the Delta (B.1.617.2) variant was not only detected but also collected and fully sequenced in various locations across multiple continents, including North America, South America, Europe, Asia, Africa, and Oceania, with a total of 275 genome records. Notably, 254 of these contained the nsp12:G671S mutation. During the subsequent period from April 26, 2020 to April 26, 2021, a total of 12,157 Delta genomes were collected and sequenced, of which 11,135 (92 %) also carried the nsp12:G671S substitution. A pronounced and rapid increase in the number of Delta genomes began only in mid-May 2021, marking the onset of its global expansion. The accompanying details and contextual events are described in “The first tide of nsp12:G671S”.

*The second tide of the nsp12:G671S*

The SARS-CoV-2 variants with the mutations nsp13:P77L, nsp14:A394V and nsp12:G671S, which were discovered almost simultaneously and dominated at the peak of the first nsp12:G671S tide, completely disappeared in early 2023 (Fig. 1a). However, in contrast to nsp13:P77L and nsp14:A394V, which have not been detected since then, nsp12:G671S reappeared about six months later and once again reached a 100 % prevalence. This time, its rise almost coincided with the emergence of a new mutation, nsp13:S36P, which peaked at 89 %. The details and illustrations are provided in Supplementary Material 3 (Fig. S2).

We analyzed two sets of SARS-CoV-2 genome samples collected worldwide. The first set (499 samples) was collected on January 1, 2023, during the period of nsp12:G671S frequency increase, and the second set (1,497 samples) was collected in August 2023, at the onset of its frequency decline. During the rise, almost all samples (91 %) belonged to the XBB lineage (from XBB.1 to XBB.9), with the largest fraction (39 %) corresponding to XBB.1.5. During the decline, eight months later, the set of genomes was represented by XBB.* (31 %), EG.* (30 %), FL.* (9.3 %), GK.* (3.2 %), and several other sublineages. However, almost all of them were descendants of earlier XBB, and XBB itself was derived from Omicron, not Delta, which was the carrier of the nsp12:G671S mutation during the tide 1. Thus, it appears that in the tide 2 the nsp12:G671S emerged de novo. The cause of the subsequent extinction of all these variants was the emergence of the JN.1(24A) lineage, which appeared at the end of 2023 and took over almost the entire population by early 2024.

Additionally, within the considered period, another group of three mutations (nsp12:Y273H, nsp13:M233I, and nsp13:N268S) emerged and began to spread rapidly, coinciding with the rise of the nsp12:G671S + nsp13:S36P combination (Fig. 1a, b). Globally, the mutations nsp12:Y273H (a total of 493,881 samples), nsp13:M233I (489,239) and nsp13:N268S (377,576) showed similar dynamics and in most cases appeared in genomes together. This trio therefore represents a somewhat less extensive, but still substantial process: at one point, over 40 % of all genomes carried all three mutations. Variants harboring these substitutions mainly belonged to the BQ.1.* lineage.

*The third tide of the nsp12:G671S, in progress*

In autumn 2024, the frequencies of the nsp12:G671S and nsp13:S36P mutations declined for the second time to only a few percent, dropping below 1 % by the end of the year. However, this line did not disappear completely and continued to persist at low levels through late 2024. At the beginning of 2025, for the third time, the proportion of nsp12:G671S and nsp13:S36P in the population began to grow again, reaching 50 % in July 2025. The dynamics of nsp13:S36P in 2025 completely coincides with that of nsp12:G671S, i. e. these mutations occurred mostly together during this period. This pattern suggests that, unlike the disappearance of the carriers of the nsp12:G671S mutation after the first tide, it persisted after the second tide, albeit in small numbers in individual variants and then, under favorable circumstances and/or after advantageous mutations, began to spread again.

The analysis of samples collected in early 2025 showed that the vast majority of them originated from the JN.1 variant, which gave rise to such lineages as NB.1(24B), NB.1.8.1(25B), LP.8.1(25A), KP.3.1.1(24E), XFG(25C), LF.7(24H), XEС(24F) and XDV (24D), as well as to the recombinants XDA and XEV. Among them, the largest number of genomes carrying the nsp12:G671S mutation (42 %) corresponded to the lineage NB.1.8.1 (clade 25B) and 35 %, to PQ.1–PQ.9 (also clade 25B). Since JN.1 is a descendant of XBB, the suggestion that the nsp12:G671S and nsp13:S36P mutations have been preserved and their proportion in the population has grown again appears quite plausible.

*Overview of all three tides of nsp12:G671S*

At the start of both tide 1 and tide 2, a rapid and significant change in the proportion of nsp12:G671S in the population occurred, accompanied by the emergence of new co-occurring mutations in the RTC genes (and sometimes in the rest of the virus genome) or noticeable changes in their mutation frequencies. This trend also holds for tide 3 due to nsp13:S36P, which co-occurred with nsp12:G671S in 2025 (Fig. 1a). Additionally, we identified a recent mutation, nsp12:D284Y, which accompanied the two mutations and exhibited the same frequency dynamics in 2025. It is not included in Table 1,because its prevalence among all genomes in GISAID in the time span from December 2019 to mid-July 2025 is below 1 %. However, in 2025, the frequency of this mutation followed the same pattern as nsp12:G671S and nsp13:S36P, reaching 50 % by mid-July 2025.

In addition, we noticed that during tide 3 (from early 2025 to mid-July 2025), the frequency curves of nsp12:G671S and nsp9:T35I were in antiphase (Fig. 1a). As the frequency of nsp12:G671S increased, that of nsp9:T35I decreased, and together, they consistently accounted for ≥80 % of all genomes in the weekly global samples. Based on these observations, a general trend can be inferred: when the fraction of nsp12:G671S mutation in the population changes rapidly, an emergence of new mutations in the RTC genes or changes in the frequencies of existing ones becomes more likely.

In contrast to nsp12:G671S, there are mutations with no obvious relation to others. One of them, nsp9:T35I, which emerged shortly after the onset of tide 2, displayed a nearly linear increase in frequency, from 0 to 100 %, in the interval between early 2023 and early 2024. This rise was much slower than any other curve in Figure 1, and it showed no correlation with any of them, which is quite unusual. The analysis of the genome samples carrying this mutation revealed the following. At the beginning of the growth phase in early 2023, it was represented by 51 % of FL.*, 25 % of XBB.1.9*, 20 % of EG.*, and 4 % others, including XCC recombinants. At the beginning of the decline phase in early 2025, the distribution shifted to its descendants: 29 % LP.*, 21 % XEC.*, 12 % NY.*, 7 % LP.*, 7 % MC.*, and the remaining 23 % comprised other lineages, including various recombinants: XFL, XFJ, XFH, XFC, XFB, XEW, XER, XEQ, and XEK.

**Specific properties of the considered mutations in the RTC nsp7–nsp14 proteins**

Each single mutation in amino acid sequences encoded by nucleotide triplets can include one to three changes within the triplet. Due to the degeneracy of the genetic code, there may be synonymous substitutions in RNA/DNA that do not change the amino acid, but even such mutations can have a noticeable effect on the fitness of their carrier, as shown by Shen et al. (2022). They came to this conclusion by experimenting with yeast, but they found “no particular reason why their results would not generalize to other organisms”

From the list of the previously considered mutations, we selected those with peak proportions ≥25 % of the population. By comparing their nucleotide and amino acid sequences in the reference genome and after mutation, we determined which mutated triplets appeared in the initial states and how they changed after mutation (Table 2).

**Table 2.**
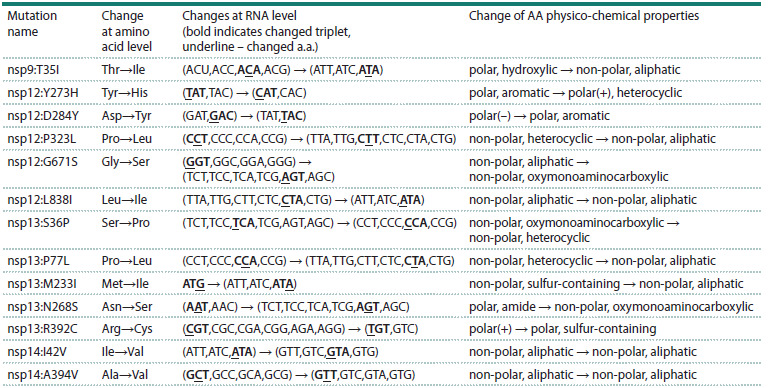
Properties of most prevalent mutations in RTC genes (peak frequency ≥ 25 %) Note. Substitutions of amino acids, changes in their physico-chemical properties and changes in RNA triplets encoding amino acids before and after mutations, are presented. In RNA triplets, the original combinations (among possible variants due to degeneracy of genetic code) are shown in boldface; changed points are underscored.

All 13 mutations examined involved only a single nucleotide substitution within the codon encoding the affected amino acid; no double or triple substitutions were detected. This is not surprising, since if p is the probability of a single rare random event, then the probability that another similar event with the same probability occurs within the two adjacent nucleotides is ~p2 or less. The probability of a single point nucleotide substitution in SARS-CoV-2 is quite low since its replication mechanism includes a proofreading system. According to data from (Amicone et al., 2022), during one cell infection cycle (i. e., from virus entry into a cell until the release of new virions), on average, 1.3 · 10−6 ± 0.2 · 10−6 substitutions occur per nucleotide position. The actual number of observable cases of 2 substitutions at once will be less than p2 because there will be only those variants which leave the virus viable despite caused changes in the replication-transcription complex, and the probability of this appears to be so small that it is practically unlikely to occur even over years. Nevertheless, it was not obvious in advance that all mutations would consist only of single-nucleotide substitutions, so this observation also brings some new knowledge of mutation patterns. However, changes can accumulate sequentially over multiple generations, experiencing only up to one nucleotide substitution per triplet in each replication cycle.

Additionally, the amino acid substitutions derived from the mutations examined frequently result in pronounced alterations of physicochemical properties

**The groups of co-existing mutations in the RTC nsp7–nsp14 proteins**

Since we noticed co-occurring mutations in RTC proteins, we decided to identify all such groups in an attempt to reveal the underlying processes and possible connections between them. Using GISAID web interface queries (as described in “Data sources”, section Materials and Methods) for the time interval from December 24, 2019 to July 15, 2025, we obtained the numbers of genome sequences containing all possible pairwise combinations of the mutations from Table 2, with the exception of nsp12:P323L (because it is present in 99.3 % of all sequences in GISAID). The results are presented in Table 3.

**Table 3.**
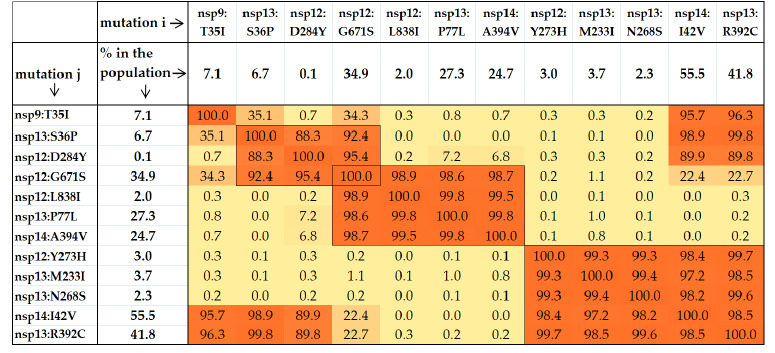
Pairs and larger groups of simultaneous mutations in RTC proteins Note. The percentage of genomes in the GISAID (collected from December 2019 to July 15, 2025) that simultaneously contain mutation i and mutation j, relative to the minimum value between the percentages of genomes with mutation i and genomes with mutation j.

For a pair of mutations, for example, i = nsp12:G671S (34.9 % of total genomes) and j = nsp13:P77L (27.3 % of all genomes), the value in cell [i][j] of the colored matrix corresponds to 98.6 % of the smaller of the two proportions (34.9 and 27.3 %). Thus, the number of genomes carrying both mutations simultaneously is approximately 0.273 ∙ 0.986 ∙ 15.5 million ≈ 4.2 million. In the global dataset, the most frequent pairs include:

– nsp13:R292C + nsp14:I42V (41.2 %), – nsp12:G671S + nsp13:P77L (26.9 %), – nsp13:P77L + nsp14:A394V (24.7 %), – nsp12:G671S + nsp14:A394V (24.4 %). Extending to triplets, representative high-frequency sets (among the 15.5 million GISAID genomes analyzed) are: – nsp12:G671S + nsp13:P77L + nsp14:A394V (24.3 %), – nsp13:R392C + nsp14:I42V + nsp13:M233I (3.6 %), – nsp13:R392C + nsp14:I42V + nsp12:Y273H (2.9 %).

A notable triplet here is nsp12:G671S + nsp13:S36P + nsp12:D284Y (0.08 %), as nsp12:D284Y emerged only in 2025, and the number of genome samples carrying this mutation continues to increase, with its peak frequency reaching 50 % (see “The second tide of the nsp12:G671S”, section Results and Discussion).

Finally, there are groups of genome samples with four or five mutations occurring simultaneously. Illustrative sets include: – nsp13:R392C + nsp14:I42V + nsp13:M233I + nsp12:Y273H: (2.89 %), – nsp13:R392C + nsp14:I42V + nsp13:M233I + nsp12:Y273H + nsp13:N268S: (2.23 %).

**Three-dimensional visualization of the selected mutations**

**Fig. 3.**
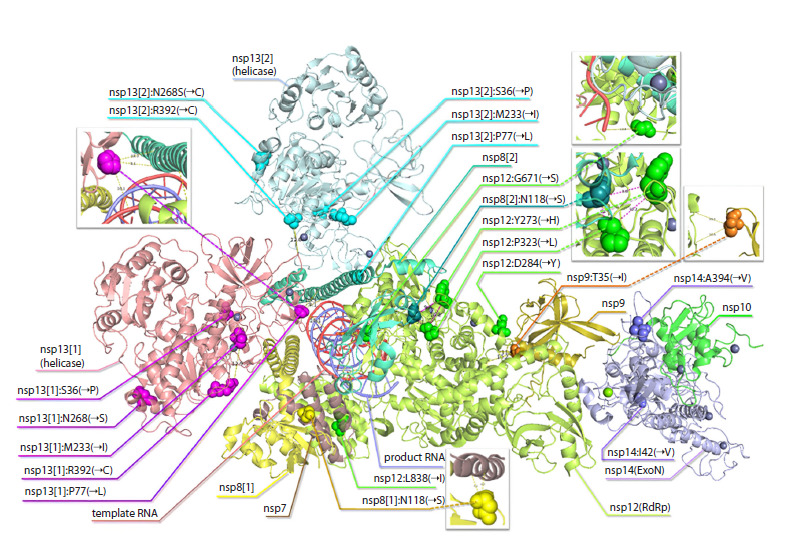
3D structure of the RTC, and its amino acids with mutations under consideration shown. Distribution of the mutations from Table 3 over the replication-transcription complex (PDB:7EIZ) including nsp7, 2×nsp8, nsp9, nsp10, RdRp(nsp12), 2×nsp13, and nsp14 together with template RNA and product RNA. Cases with specific features are shown in the insets. The generated PyMOL files based on PDB RTC 3D structures and examined mutations, are available upon request

We rendered the examined mutations listed in Table 2 in the 3D structure of the RTC to learn about their spatial location, specific features, and distances between mutated amino acids, as well as between amino acids and RNA threads (Fig. 3).

According to Figure 3, the highest concentration of mutations is observed in nsp9 and nsp13 (helicase): 0.88 and 0.83 substitutions per 100 amino acids, respectively. Half as many mutations, 0.43 and 0.38 per 100 aa, occur in nsp12 (RdRp) and nsp14 (ExonN), correspondingly. In the remaining proteins, no mutations with a peak frequency exceeding 25 % of the population size were found. Three of four shown mutations in nsp12 (RdRp) are located on the surface of the polymerase, on the side closer to RNA. There are several proximity-based (within ~6–14 Å) potential relationships that may merit functional follow-up. The mutated amino acid closest to the template RNA (9.1 Å) is nsp13:M233I (Fig. 3, inset). In nsp13, P77L is positioned 10.1 Å from the RNA strand coursing through the complex and 9.1 Å from an nsp8 α-helix, placing it in a location where effects on replication kinetics and/or fidelity are plausible. Next, nsp8:N118S resides 6.1 Å from nsp7, which may affect the interaction between them

RTC includes two copies of nsp8 and two copies of nsp13, with different neighborhoods for each protein of a couple. In one nsp8, the amino acid N118 (mutation N→S) lies near two nsp12 amino acids corresponding to the P323L and Y273H mutations: the distance between N118 and P323 is 5.1 Å, between N118 and Y273 is 9.8 Å, and between P323 and Y273 is 10.2 Å. This triangle (Fig. 3, inset) constitutes a cluster of three amino acids involved in mutations, close to each other, and we flag this cluster as a priority for subsequent functional investigation.

**Search for literature on SARS-CoV-2 RTC mutation effects**

To complement our mutational analysis, we performed a literature search for all substitutions listed in Table 2. The goal was to identify any previously reported structural, biochemical, or phenotypic effects associated with these mutations. Brief summaries of the available evidence are provided in Supplementary Material 4. Overall, the collected publications describe diverse potential impacts, including changes in RTC stability, replication efficiency, drug response, and enzymatic activity, although for many mutations experimental validation remains limited.

## Conclusion

We surveyed the evolution of the SARS-CoV-2 RTC (nsp7– nsp14) over a 5.5-year horizon (December 2019 – July 15, 2025), identified 22 amino acid substitutions with a global average frequency >1 % and quantified their weekly dynamics. Compared to earlier global overviews that used higher prevalence thresholds (Rodriguez et al., 2023, 2025), this extended window and finer temporal resolution revealed three characteristic dynamic patterns: persistent, transient, and recurrent. Interestingly, one notable mutation, nsp12:G671S, exhibited a unique multi-phase trajectory (“three tides”) marked by a rise, near-disappearance, and re-emergence.

Re-analysis of early records indicated that SARS-CoV-2 genomes deposited to GISAID database and assigned to Delta (B.1.617.2) variant have submission dates that are 7–8 months earlier than commonly reported date of the first appearance of Delta. GISAID’s first samples of Delta appear for the first time on April 26, 2020, they spanned multiple continents by the end of 2020 (and frequently carrying the trio of mutations nsp12:G671S + nsp13:P77L + nsp14:A394V). This observation motivates a reassessment of the widely cited timeline stating that “Delta was first identified in Maharashtra, India, in late 2020” (Cherian et al., 2021) and emphasizes the value of stringent filtering of public surveillance datasets

In the course of systematically scanning RTC variation, we revealed a robust linkage between Delta and the nsp12:G671S + nsp13:P77L + nsp14:A394V trio. Tracing these substitutions back showed that each appeared separately before Delta, and that early genomes labeled as Delta appeared sporadically for several months but had little immediate effect on global frequency among sequenced genomes. The subsequent co-occurrence of all three substitutions around August 2020 coincided with a marked shift to rapid expansion and, soon after, global dominance. This perspective emerges specifically from an RTC-centered analysis (traditionally viewed as a conserved, lower-visibility target) and, in our view, illuminates otherwise overlooked contingencies in the evolutionary dynamics of the virus.

Across waves, nsp12:G671S re-emerged with different companions: first with nsp13:P77L and nsp14:A394V, later with nsp13:S36P, and most recently with nsp12:D284Y. In contrast, nsp9:T35I followed a slow, largely independent, monotonic rise, longer period of 97–100 % prevalence, and a slow decline that began in 2025. A codon-level inspection of the 13 highest-peak substitutions (≥25 %) showed only single-nucleotide changes (no double/triple codon substitutions), yet many corresponding amino acid replacements entail substantial physicochemical shifts. Co-occurrence analysis highlighted frequent groups of co-existing mutations, ranging from two to five. Structural mapping onto RTC assemblies showed a relatively even spatial distribution without dense clusters; only nsp13:M233I had a notable feature – it lay directly adjacent to the template RNA. The repeated rise and fall in the number of genome samples with the nsp12:G671S mutation, given the accompanying events, probably represents an example of natural selection in action. We can propose as a hypothesis for discussion that it may be not a neutral change, but a key player in the evolutionary “strategy” that provides a short-term fitness advantage (compensating for proofreading errors) at the expense of long-term stability (increasing mutation load). This creates a predictable cycle of emergence and extinction – a pattern that is the hallmark of selection, not random drift.

Independent population genetic analyses have shown that the evolutionary rate of SARS-CoV-2 varies among lineages and can undergo episodic accelerations, up to fourfold exceeding the baseline phylogenetic rate, with new variants emerging within weeks rather than months (Tay et al., 2022). The recurrent G671S tides with changing co-mutation partners documented in this study are consistent with such episodic phases. Given that several high-prevalence co-mutations lie in nsp14 (ExoN), these patterns raise the question of whether fluctuations in effective error correction could accompany periods of rapid diversification. Targeted assays will be required to test this hypothesis

To contextualize these findings, we reviewed the available literature on all substitutions from Table 3 (Supplementary Material 4). Reported effects encompass altered polymerase stability/transmissibility for nsp12:P323L/G671S (Kim et al., 2023), lineage-linked occurrences and additional RTC changes documented through mid-2023 (Rodriguez et al., 2023, 2025), and mixed evidence regarding drug response and enzymatic activity for several sites. While suggestive, many substitutions still lack a definitive experimental validation of their functional impact. Protein-protein and protein-RNA interactions are complicated, and they involve many factors even if a single protein is considered (for example, (St Laurent et al., 2012, Fig. 5)). Our study is focused on one of the most intricate protein complexes with a critically important function, RNA replication.

To sum up, our results refine the timeline of early Delta emergence and dissemination, document repeated large-scale shifts centered around nsp12:G671S, and provide a reproducible catalog of RTC mutation dynamics, co-occurrence, coding changes, and structural context. The continued integration of high-quality genomic surveillance with targeted biochemical and virological assays will be essential to resolve the functional consequences of these substitutions and to anticipate future shifts in the RTC mutational landscape.

## Conflict of interest

The authors declare no conflict of interest.
